# *N*-acetyl aspartate concentration in the anterior cingulate cortex in patients with schizophrenia: A study of clinical and neuropsychological correlates and preliminary exploration of cognitive behaviour therapy effects

**DOI:** 10.1016/j.pscychresns.2010.02.008

**Published:** 2010-06-30

**Authors:** Preethi Premkumar, Vivek A. Parbhakar, Dominic Fannon, David Lythgoe, Steven C. Williams, Elizabeth Kuipers, Veena Kumari

**Affiliations:** a Department of Psychology, Institute of Psychiatry, King's College London, London, United Kingdom; b Division of Psychological Medicine and Psychiatry, Institute of Psychiatry, King's College London, London, United Kingdom; c Centre for Neuroimaging Sciences, Institute of Psychiatry, King's College London, London, United Kingdom; d NIHR Biomedical Research Centre for Mental Health, South London and Maudsley NHS Foundation Trust, London, London, United Kingdom

**Keywords:** Chemical shift imaging, Cognitive behaviour therapy, Neuropsychological function, Magnetic resonance spectroscopy, Metabolism, Symptoms

## Abstract

This study investigated the clinical and neuropsychological correlates of *N*-acetyl aspartate (NAA) concentration in the anterior cingulate cortex (ACC) in schizophrenia, and explored whether ACC NAA concentration is sensitive to symptom change following cognitive behaviour therapy for psychosis (CBTp). Participants comprised 30 patients and 15 healthy controls who underwent magnetic resonance spectroscopy of the ACC and were assessed on frontal lobe based neuropsychological tasks. Twenty-four (of 30) patients were followed-up; 11 subsequently received 8–9 months of CBTp in addition to standard care (CBTp + SC) and 13 received SC only. At baseline (i) NAA and Cr concentrations were lower in patients compared to controls, (ii) in patients, NAA concentration correlated inversely with positive symptoms and general psychopathology (positive symptoms explained 21% of the variance; total variance explained = 25%) and Cho concentration correlated inversely with positive symptoms, and (iii) in controls, NAA concentration correlated positively with working and short-term memory and Cr concentration inversely with executive function. NAA concentration tended to increase in CBTp + SC patients at follow-up (*n* = 7 with usable data) concomitant with improvement in positive symptoms. NAA concentration may be more closely associated with symptoms and symptom change than frontal lobe based neuropsychological function in schizophrenia, perhaps because the latter is relatively stable during the long-term illness course.

## 1 Introduction

Schizophrenia is often characterised by a profile of clinical and neuropsychological impairment that is consistent with a frontal lobe based pathology (Weinberger et al., 1994). Structurally, grey matter (GM) reduction in the anterior cingulate cortex (ACC) has been observed in patients with schizophrenia (Baiano et al., 2007; Fornito et al., 2008), though such reductions are not limited to the ACC (see Honea et al., 2005, for a meta-analytic review of GM reductions in schizophrenia).

A further mode of investigating the neuropathology of schizophrenia is at the neurochemical level. *N*-acetyl aspartate (NAA) loss and impaired mitochondrial function are characteristic of several neurodegenerative diseases, including Alzheimer's Disease and multiple sclerosis (Perry et al., 2002; Rutten et al., 2003). These findings suggest that NAA concentration may be an indicator of mitochondrial function. *N*-methyl D-aspartate (NMDA) receptor activation coupled with Ca2+ cellular influx has been reported to increase neuronal release of NAA and delay nerve cell death (Tranberg et al., 2004). NAA concentration thus may also be an indicator of NMDA function and neuronal longevity. Interestingly, NMDA glutamate receptor dysfunction has been suggested to sub-serve many abnormalities associated with schizophrenia (Moghaddam, 2003). It has been reported that inhibitors of NMDA function, such as phencyclidine or ketamine, precipitate symptoms of schizophrenia in healthy people and exacerbate symptoms in patients with schizophrenia (Adler et al., 1999; Krystal et al., 1994; Lahti et al., 1995; Malhotra et al., 1997). Ketamine is also found to cause disruptions in working memory in both healthy and schizophrenia populations (Adler et al., 1999), indicating that deficient activation of NMDA receptors may be crucially involved in neuropsychological deficits commonly observed in patients with schizophrenia. Choline (Cho) and creatine/phosphocreatine (Cr) concentrations may also be important to neuronal integrity. Cho is involved in the synthesis of membrane phospholipids (for review, see Ross and Sachdev, 2004), while Cr helps maintain the energy homeostasis within the nerve cell (Andres et al., 2008). The evidence suggests that Cr may also have direct effects on neuropsychological function. For example, oral supplements of Cr increased intelligence, working memory performance (Rae et al., 2003) and executive function (McMorris et al., 2007, 2006).

Previous studies have reported, using magnetic resonance spectroscopy (MRS), lower ACC NAA level in patients with schizophrenia, especially in those treated with typical antipsychotic drugs, compared with healthy participants (Braus et al., 2002; Deicken et al., 2000; Ende et al., 2000; Sanches et al., 2008; Tayoshi et al., 2009; Wood et al., 2007; Yamasue et al., 2002; see Appendix A for detailed information). Within this patient population, ACC NAA concentration is reported to be inversely associated with illness duration in some studies (Braus et al., 2002; Ende et al., 2000; Theberge et al., 2003), but not others (Deicken et al., 1997; Tayoshi et al., 2009; Theberge et al., 2004; Wood et al., 2007; Yamasue et al., 2002). NAA reductions have been reported in antipsychotic drug-naïve first-episode patients, and treatment may increase NAA levels in both recent-onset cases and patients with chronic illness (Braus et al., 2002; Fannon et al., 2003). NAA concentration is positively associated with duration on atypical antipsychotic medication in long-term patients (Braus et al., 2002), and inversely correlated with the duration of positive symptoms in first-episode neuroleptic-naïve patients (Theberge et al., 2004). More recently, Tayoshi et al. (2009) observed no association between NAA concentration and symptoms, but reported a positive association between NAA concentration and Wisconsin Card Sorting Test (WCST) performance; the latter finding was also present in two earlier studies (Braus et al., 2002; Ohrmann et al., 2008).

Neuropsychologically, the ACC is involved in components of the central executive that facilitate working memory (Osaka et al., 2004; Smith and Jonides, 1999), including encoding of semantic information (Kaneda and Osaka, 2008), and reported to be deactivated in schizophrenia patients compared with healthy participants during the stimulus encoding phase of a working memory task (Schlosser et al., 2008). A further role of the ACC is in emotional decision-making, where there is a relationship between an action and the reinforcement value of its outcome (Rushworth et al., 2004; Walton et al., 2007). The ACC is involved in updating and integrating recent information to existing information on reward value of outcome in terms of predicting future outcome (Behrens et al., 2007; Kennerley et al., 2008).

The present study has two parts: cross-sectional and longitudinal. Cross-sectionally, the study aimed to compare the ACC NAA concentrations in patients with schizophrenia and healthy participants, and to determine their clinical and/or neuropsychological correlates; it also explored these effects in Cho and Cr. All except for three recent studies that examined these metabolites in patients with schizophrenia (Ohrmann et al., 2008; Sanches et al., 2008; Tayoshi et al., 2009) had a smaller sample size than that in our investigation, and none undertook a comprehensive examination of metabolite concentration relationships with clinical symptoms and neuropsychological functions. We hypothesized that (1) NAA concentration would be reduced in patients compared with healthy participants due to impaired neuronal function; (2) in patients, NAA concentration would be associated inversely with illness duration (Braus et al., 2002; Ende et al., 2000; Theberge et al., 2003) and symptoms on the Positive and Negative Syndrome Scale (Wood et al., 2007); and (3) NAA concentration would be positively associated with neuropsychological measures that involve the ACC, namely working memory, conflict detection and decision-making (Behrens et al., 2007; Carter and van Veen, 2007; Osaka et al., 2004; Peterson et al., 1999; Smith and Jonides, 1999; Walton et al., 2007), both in healthy participants and patients, though such relationships may be weaker in patients because they, as mentioned earlier, engage the ACC at a less-than-normal level during relevant tasks. We expected that the clinical or neuropsychological correlates of metabolite concentration would be attenuated or abolished after controlling for ACC grey matter (GM) concentration (Yamasue et al., 2002), as GM concentration may influence metabolite availability.

Longitudinally, the study aimed to determine the effect of cognitive behavioural therapy for psychosis (CBTp) on metabolite concentration. A sub-sample of the patients in the present investigation went on to receive CBTp in addition to their standard care. The study therefore also examined whether (i) baseline metabolite concentration predicted symptom change following CBTp + SC and (ii) there was a change in metabolite concentration in patients who received CBTp in addition to standard care. We hypothesized that in this sub-sample, NAA concentration would increase coincident with a reduced level of symptoms following CBTp.

## 2 Materials and method

### 2.1 Participants and design

The study involved 30 patients with schizophrenia and 15 healthy participants, all of whom underwent MRS of the ACC at study entry. The patient and control groups were matched on average for age and gender (Table 2). All patients were assessed by a Consultant Psychiatrist (DF) for clinical diagnosis using the Structured Clinical Interview for DSM-IV Axis I disorder (First et al., 2002) and symptom severity using the Positive and Negative Syndrome Scale (PANSS, Kay et al., 1987). All patients were recruited from the South London and Maudsley NHS Foundation Trust as part of a larger project investigating the functional neuroimaging correlates and predictors of CBTp (the MRS component was added to the larger project), had been on stable doses of antipsychotic medication for at least 2 years, and on the current antipsychotic drug for at least 3 months before taking part.

Of the 30 patients at study entry (baseline), 11 patients subsequently received CBTp + SC in a specialist clinical service [Psychological Interventions Clinic for Outpatients with Psychosis (PICuP), South London and Maudsley NHS Foundation Trust (SLAM)] in addition to their standard care, and 13 patients continued to receive standard care only (SC controls); all of these 24 patients (i) received a rating of ≥ 60 on the PANSS, (ii) reported at least one positive ‘distressing’ symptom of schizophrenia, and (iii) wished to receive CBTp (if available: only about 10–20% of eligible SLAM patients were accepted for CBTp with the existing resources; availability was the only criterion that determined whether or not eligible patients received CBTp). Eight of 11 CBTp + SC patients and 12 of 13 SC control patients were assessed for symptom severity at follow-up, of whom seven and four patients, respectively, underwent MRS at follow-up. Six (of 30) patients had low symptom severity at baseline; they were considered to be responsive to antipsychotic treatment by their long-term treating clinicians (SC responsive); these patients were not followed up.

The patients in the CBTp + SC and SC control groups were recruited from the same geographical area, were identified by local Consultant Psychiatrists as suitable for CBTp, and wished to receive CBTp in addition to their usual care. Allocation to CBTp + SC and SC control groups was not randomised, but followed a cohort case-controlled design. Patients who were referred to and accepted for CBTp by the Psychological Interventions Clinic for Outpatients with Psychosis (PICuP), SLAM NHS Foundation Trust went into the CBTp + SC group. Others who matched demographically (age and number of years in education) and clinically (illness duration and PANSS symptoms) as much as possible those accepted for CBTp by the PICuP were studied as part of the SC control group over the same interval as the CBTp + SC group patients. SC consisted of case management offered by the case management team for a particular geographical area. Most patients (80%) were on a single antipsychotic drug (see Table 2). Of the 12 patients on olanzapine, one patient also took aripiprazole and a further patient also took trifluoperazine. Of the six patients on clozapine, one patient also took quetiapine and a further patient also took amisulperide. Of the two patients on haloperidol, one patient was also on sulperide and the other patient on pipotiazine. In addition to antipsychotic drug treatment, four patients were receiving anti-cholinergics.

CBTp followed the procedure developed by Fowler et al. (1995). Therapy sessions were conducted on a weekly or fortnightly basis, as preferred by the patient. Patients received an average of 16 sessions. All CBT interventions were formulation-driven, and focused on the therapy goals of the patient. The therapists were supervised by one of the investigators (EK) who has extensive experience of CBTp.

The study procedures were approved by the ethics committee of the Institute of Psychiatry and the South London and Maudsley NHS Foundation Trust, London. All participants provided written informed consent to their participation and were compensated for their time and travel.

### 2.2 Neuropsychological assessments

All patients and 13 healthy participants were assessed on neuropsychological measures (described in Table 1) that are considered to recruit the ACC and found to reveal performance deficits in executive function, working/short-term memory, attention and emotional decision-making in schizophrenia.

### 2.3 Magnetic resonance spectroscopy: data acquisition and processing

Images were acquired using a 1.5 Tesla GE N/Vi Signa System (General Electric, Milwaukee, WI, USA) at the Maudsley Hospital, South London. A quadrature birdcage head coil was used for RF transmission and reception. Initially, a series of fast gradient echo scout images were acquired in order to orient subsequent images relative to the anterior commissure/posterior commissure line and the interhemispheric fissure. Subsequently, the whole brain was scanned with a 3-D inversion recovery prepared fast spoiled GRASS T1-weighted dataset. Chemical shift images were acquired from a PRESS excited volume in a 1.5 cm thick slab through the anterior commissure, corresponding with the dorsal ACC, with a nominal voxel size of 1.5 cm^3^ (24 × 24 phase-encode steps over a 24 cm FOV). To minimise relaxation effects, a short TE of 35 ms was used, and a relatively long TR of 2 s.

**Fig. 1:**
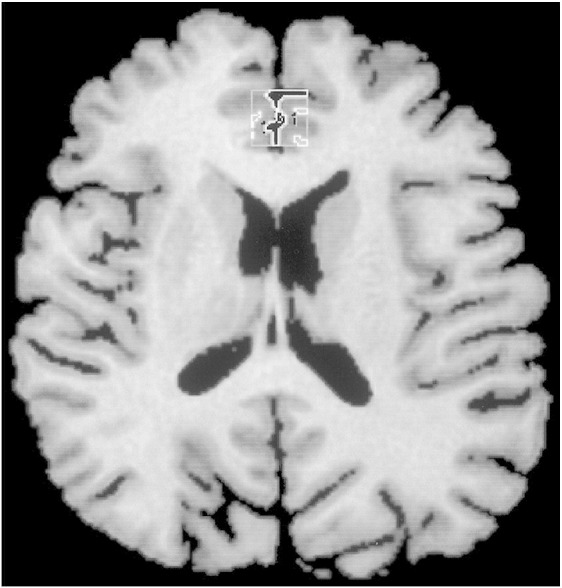
A Spoiled Gradient Recalled (SPGR) brain image in the axial slice in a control subject. The square indicates the volume of interest (VOI), 1.5 × 1.5 × 1.5 cm^3^ voxel in the anterior cingulate cortex. Within the VOI, each tissue (cerebrospinal fluid, grey matter and white matter) was segmented.

Only metabolite information with a fitting error (percent standard deviation) of < 20% was included in the final analysis (Ohrmann et al., 2008). Absolute concentrations of NAA, Cho and Cr were measured in millimolars in all participants using the LC model (McLean et al., 2000; Provencher, 1993). For each MRS voxel, the proportions of GM, white matter and cerebrospinal fluid (CSF) were calculated by segmenting the Spoiled Gradient Recalled (SPGR) image (Fig. 1). This allows correction for CSF contamination within each voxel and extrapolation to concentrations for pure GM (McLean et al., 2000). Metabolite concentration was calculated as a function of CSF concentration within each voxel using the following formula,

$$\text{Metabolite value}/\frac{(1-\text{CSF concentration)}}{100}$$

NAA, Cho and Cr absolute concentration estimates in millimolars are reported rather than metabolite peak ratios, such as NAA/Cho, NAA/Cr and Cho/Cr, because absolute concentrations of individual metabolites are not influenced by fluctuations in the concentration of the reference metabolite. Metabolite ratios are believed to account for alterations in the Cr reference peak which invalidate its usefulness as an internal reference for the other metabolite peaks of the proton spectrum (Deicken et al., 1997).

### 2.4 Statistical analysis

#### 2.4.1 Cross-sectional analysis

##### 2.4.1.1 Group comparison of metabolite concentration

Differences between patient (across all patients) and healthy participant groups in metabolite concentration and GM concentration, age, years of education, and clinical and neuropsychological variables were examined using analysis of variance (ANOVA). For the neuropsychological variables showing a non-normal distribution (Brixton perseverative errors), Mann–Whitney *U* tests were performed. A chi-squared test examined group differences in gender distribution.

##### 2.4.1.2 Clinical correlates of metabolite concentration

Pearson and partial correlations controlling for GM concentration, or Spearman correlations for clinical variables with a non-normal distribution (antipsychotic medication level), were performed between clinical variables and metabolite concentrations. The clinical variables were PANSS symptoms, illness duration (defined as the difference between the age at onset of psychotic symptoms as reported by the patient and where possible confirmed with other sources and age at the time of baseline assessments) and antipsychotic medication level (in chlorpromazine equivalents). Spearman correlations were performed to examine the relationship between metabolite values and individual PANSS items in those subscales that were found to have a significant association with metabolite values.

##### 2.4.1.3 Neuropsychological correlates of metabolite concentration

Pearson and partial correlations controlling for GM concentration, or Spearman correlations for neuropsychological variables with a non-normal distribution (Brixton perseverative errors), were performed between neuropsychological performance measures and metabolite concentration in patient and healthy participant groups.

##### 2.4.1.4 Multiple regression of clinical and/or neuropsychological correlates on metabolite concentration

Significant clinical and/or neuropsychological variables that had a normal distribution were entered into the multiple regression model using a stepwise method (to estimate the effect of individual predictors on variability of criterion variable) and using a forced-entry method (to explain the combined effect of predictors on the variability of the criterion variable).

#### 2.4.2 Longitudinal analysis

##### 2.4.2.1 Group comparison of metabolite concentration

Due to the small sample sizes, differences between CBTp + SC, SC control and SC responsive patient groups in age, education, clinical variables, and baseline metabolite and GM concentrations were examined using non-parametric analyses, namely the Kruskal-Wallis test. A chi-squared test determined group differences in gender distribution.

##### 2.4.2.2 Metabolite concentration as a predictor of symptom change following CBTp

Spearman correlations were performed in the CBTp + SC patients who were followed up (*n* = 8) between baseline metabolite concentrations and residual PANSS symptom change scores from baseline to follow-up.

##### 2.4.2.3 Change in symptom severity and metabolite concentration following CBTp

Due to the small sample sizes (CBTp + SC, *n* = 7; and SC control, *n* = 4), changes in symptom severity, metabolite and GM concentrations from baseline to follow-up in the patient groups were examined using the Wilcoxon Signed Rank test.

All analyses were performed in SPSS windows (version 15). Alpha level for testing significance of effects was maintained at *P* < 0.05. We chose not to apply the Bonferroni method of adjusting the *P* value because we expected the size of the relationship between MRS values and symptoms/neuropsychological functions to be small or at best modest given that symptoms in schizophrenia are unlikely to have a single aetiology and successful task performance on most neuropsychological tasks would recruit additional brain regions.

## 3 Results

### 3.1 Cross-sectional analysis

#### 3.1.1 Group differences in demographic and neuropsychological variables

Patient and healthy participant groups did not differ in gender and age. Patients had fewer years in education and showed deficient performance on most neuropsychological measures relative to healthy participants (Table 2).

#### 3.1.2 Group differences in metabolite concentration in the ACC

NAA, Cr and GM concentrations were lower in the patient compared with the healthy participant group (Table 3).

#### 3.1.3 Clinical correlates of metabolite concentration

NAA concentration correlated inversely with positive symptoms, general psychopathology and total symptoms (Table 4). These correlations remained significant after controlling for GM. NAA concentration correlated inversely with negative symptoms only after controlling for GM. PANSS item-wise correlations revealed that NAA concentration was inversely correlated with delusions, disorganisation and persecution items of the positive syndrome subscale, passive social withdrawal and stereotyped thinking items of the negative syndrome subscale, and somatic concern and preoccupation items of the general psychopathology subscale.

Cho concentration correlated inversely with positive and total symptoms. After controlling for GM concentration, Cho concentration correlated with total symptoms, but not positive symptoms. Cho concentration correlated inversely with negative symptoms at a trend level only after controlling for GM concentration. PANSS item-wise correlations revealed that Cho concentration was inversely correlated with disorganisation and hostility items of the positive syndrome subscale, passive social withdrawal item of the negative syndrome subscale, and somatic concern item of the general psychopathology subscale. Cho concentration correlated positively with antipsychotic medication dosage. Cr concentration did not correlate with any clinical variable.

#### 3.1.4 Neuropsychological correlates of metabolite concentration

*Patients*: A positive correlation between NAA concentration and working memory as assessed by the letter number test emerged only after controlling for GM concentration (Table 5).

*Healthy participants*: NAA concentration correlated positively with working memory as assessed by the letter number test and short-term memory as assessed by the Hopkins verbal learning test, though these correlations were reduced to a trend level of significance after controlling for GM concentration (Table 5). Cho concentration correlated inversely with short-term memory as assessed by the Wechsler Memory Scale—Revised (WMS-R) logical memory immediate recall test after controlling for GM concentration. Cr concentration correlated inversely with executive function as assessed by Brixton perseverative errors.

#### 3.1.5 Multiple regression of clinical and/or neuropsychological correlates of metabolite concentration

*Patients*: In the stepwise regression model with NAA concentration as the criterion variable, positive symptoms proved to be a significant predictor (*r* =−0.489, *P* = 0.006). The model explained 21% of the variance in NAA concentration. In the forced-entry regression model, the predictors (positive symptoms, general psychopathology and letter number test) explained 25% of the variance in NAA concentration.

*Healthy participants*: In the stepwise regression model with NAA concentration as the criterion variable, short-term memory as assessed by the Hopkins verbal learning test was a significant predictor (*r* = 0.576, *P* = 0.039). The model explained 27% of the variance in NAA concentration. In the forced-entry regression model, the predictors (Hopkins verbal learning test and letter number test) explained 27% of the model.

### 3.2 Longitudinal analysis

#### 3.2.1 Metabolite concentration differences at baseline between patient sub-groups

NAA concentration tended to differ between patient groups at baseline mainly due to greater concentration in the low-symptom SC-responsive group (Table 6).

### 3.3 Metabolite concentration correlates of symptom change following CBTp

In the CBTp + SC group, metabolite concentration at baseline was not associated with symptom change following CBTp (Supplementary Table).

### 3.4 Symptom severity and metabolite concentration change from baseline to follow-up in CBTp + SC and SC control groups

Positive symptoms improved following CBTp in the CBTp + SC group, while negative symptoms tended to deteriorate in the SC control group (Table 7).

NAA concentration tended to increase in the CBTp + SC group from baseline to follow-up (Table 7).

## 4 Discussion

The present investigation examined the concentrations of NAA, Cho and Cr in the ACC and their clinical and neuropsychological correlates in patients with schizophrenia and healthy participants (cross-sectional investigation). The study also explored whether baseline metabolite concentration predicted symptom change following CBTp + SC and whether a reduction in symptoms following CBTp + SC was accompanied by higher NAA concentration relative to baseline (longitudinal investigation).

### 4.1 Summary of clinical and neuropsychological correlates of metabolite concentration (cross-sectional investigation)

The major findings from the cross-sectional analysis were that in patients: (1) NAA concentration was reduced compared to healthy participants, (2) greater NAA concentration was associated with a lower level of positive symptoms, general psychopathology and better working memory (positive symptoms alone explained 21% of the variance; total variance explained = 25%), and (3) greater Cho concentration was associated with a lower level of positive symptoms and higher level of antipsychotic medication. In healthy participants the following associations were observed: (1) NAA concentration positively with working and short-term memory, (2) Cho concentration inversely with short-term memory, and (3) Cr concentration inversely with executive function. These findings will be discussed in turn.

#### 4.1.1 Clinical correlates of metabolite concentrations

The findings of lower ACC NAA concentration in schizophrenia patients (Deicken et al., 1997; Ende et al., 2000; Wood et al., 2007) and clinical correlates of NAA concentration support previous evidence (Theberge et al., 2003; Wood et al., 2007; Yamasue et al., 2002). Our study of a relatively larger sample of patients than most previous studies is the first to observe that NAA concentration was inversely associated with the severity of positive, negative and general psychopathology symptoms based on the three-dimensional model of the PANSS (Kay et al., 1987). Twenty percent of our patients were SC treatment responsive, giving a wider range to symptom scores than earlier studies. Patients who had low symptom severity and were considered to have responded sufficiently to their standard (mostly atypical) antipsychotic treatment tended to show greater NAA concentration than patients who were symptomatic (baseline comparisons), suggesting that NAA concentration may vary as a function of symptom severity and treatment response.

NAA concentration being a by-product of neurotransmitter turnover (Tranberg et al., 2004), the symptom severity-metabolite concentration associations may reflect the efficiency of neurotransmission. As noted in Section 1, NAA concentration may be an indicator of NMDA function and neuronal longevity (Tranberg et al., 2004). NAA efflux is a delayed response to sudden non-physiological decrease in extracellular osmolarity and high K + intra-cellular and NMDA activation (Tranberg et al., 2007). Elevated concentration of NAA in the ACC in schizophrenia patients with lower level of symptoms may reflect increased turnover of neurotransmitters such as dopamine and NMDA.

Similarly, the association between Cho concentration and total and positive symptoms may also reflect improved neurotransmitter function and neuronal integrity with milder symptoms. The Cho peak includes soluble membrane phospholipids including phosphorylcholine (PCho), glycerophosphocholine (GPCho) and a relatively negligible amount of free choline (Ross and Sachdev, 2004). PCho is involved in synthesis of the insoluble membrane phospholipids, while GPCho is a product of membrane degradation, and free choline is involved in synthesis of the neurotransmitter acetylcholine, as well as in membrane synthesis (Ross and Sachdev, 2004). An increase in the Cho peak is associated with an increase in membrane breakdown or turnover and myelination (Ross and Sachdev, 2004). The positive association between antipsychotic medication dosage and Cho concentration, a measure of neuronal membrane turnover (Miller, 1991; Ohrmann et al., 2008), suggests that medication level also influences neuronal activity.

#### 4.1.2 Neuropsychological correlates of metabolite concentration

In patients, the finding of an association between greater NAA concentration and better working memory supports evidence for the role of frontal lobe function in working memory using functional MRI and proton MRS in schizophrenia (Bertolino et al., 2000; Callicott et al., 2000; Ohrmann et al., 2007), although this association explained only a very small amount (3%) of the variance in NAA concentration that was additional to that explained by positive symptoms.

In the healthy group, greater NAA concentration was associated with better working and short-term memory. The ACC is activated during both working and short-term memory (Kim et al., 2006; Lenartowicz and McIntosh, 2005). NAA concentration in the frontal lobe white matter was positively associated with working memory in healthy children, which may be related to increased number of dendritic processes at an early age (Yeo et al., 2000). NAA concentration has a role in neuronal integrity in the form of increased myelination and mitochondrial function (Ross and Sachdev, 2004) and this may facilitate working and short-term memory.

Greater Cho concentration correlated inversely with short-term memory in the healthy group when controlling for GM concentration in the ACC. A negative association between Cho concentration in the occipito-parietal white matter and IQ, independent of the positive association between NAA concentration and IQ, in healthy individuals has been previously reported (Jung et al., 1999). Our findings suggest independent metabolite concentration-neuropsychological associations between NAA, Cho and Cr in healthy individuals. Greater Cr concentration was associated with poorer executive function in the present study. A study of elderly males found negative correlations between adjusted Cr in the parietal lobe and various cognitive measures including logical memory and verbal memory (Ferguson et al., 2002). The study did not include a measure of executive function. Ferguson et al. (2002) believed that the findings fit with evidence that higher Cr levels are associated with cognitive ageing. They proposed that poorer neuropsychological performance in healthy ageing is associated with an increase in Phosphocreatine (PCr) due to a reduction in conversion of PCr to Adenosine-tri-phosphate (ATP) for energy. Increased Cr concentration may be a marker of decreased brain energy metabolism.

### 4.2 Effect of cognitive behaviour therapy for psychosis on metabolite concentration

Our preliminary investigation of change in metabolite concentration in a small number of patients who received CBTp + SC compared to patients who received SC only revealed a trend for an increase in NAA concentration with CBTp. This increase in NAA was concomitant with improvement in positive symptoms in CBTp + SC patients. This finding is consistent with the view that NAA concentration increases with alleviation of symptoms as discussed above. Recent functional imaging studies also provide evidence for a role of the ACC in responsiveness to CBT in psychosis (Kumari et al., 2009) as well as major depression (Costafreda et al., 2009).

### 4.3 Limitations and future research

One of the limitations of the study was that the sample sizes of patients who received CBTp + SC or SC only and were clinically assessed and/or scanned at follow-up were small. A further limitation was that gender distribution differed between the patient groups. Although there was no gender effect within each patient group in the metabolite concentrations in the present study, higher ACC glutamine level in male compared to female schizophrenia patients has been previously observed (Tayoshi et al., 2009). Other effects of gender on ACC function have also been reported, for example, reduced ACC glucose metabolism in male chronic patients relative to healthy participants and female patients (Fujimoto et al., 2007). The predictive value of NAA concentration on symptom change in patients receiving psychological treatment or standard care needs to be examined in a larger sample involving a sufficient number of both men and women with schizophrenia. Additionally, the role of clinical and neuropsychological variables on ACC NAA levels in patients who improve with CBTp needs to be explored.

### 4.4 Conclusions

Higher NAA and Cho concentrations in the ACC in patients are associated with lower symptom severity. Stronger and more consistent associations between metabolite concentrations and symptom severity than neuropsychological function suggest that metabolite concentration and symptoms may co-fluctuate more strongly. Neuropsychological deficits may be more stable (Szoke et al., 2008) and not necessarily improve in parallel with symptom improvement (Hughes et al., 2003). NAA concentration may yet reflect symptom changes following CBTp and, if replicated in future studies, has the potential to provide a useful biological marker of clinical response to psychological treatments.

## Figures and Tables

**Table:** 

Publication	Participants	Measures	Results
Deicken et al. (1997)	Schizophrenia patients (*n* = 26; 9 on atypical and 17 on typical) Healthy participants (*n* = 16)	NAA, Cho and Cr, illness duration, antipsychotic medication dose	Patients had lower NAA concentration compared to healthy participants. No significant group differences or lateralized asymmetries for Cho or Cr. No significant correlations between NAA and either illness duration or antipsychotic medication dose.
Ende et al. (2000)	Schizophrenia patients (*n* = 19; 10 on atypical and 9 on typical) Healthy participants (*n* = 16)	NAA, Cho and Cr, age, illness duration.	Patients had lower NAA concentration compared to healthy participants. Patients receiving typical medication (but not atypical) had lower NAA concentration than healthy participants. NAA concentration was inversely correlated with age in patients and healthy participants, and with illness duration (corrected for age) in patients.
Braus et al. (2001; 2002)	Schizophrenia patients (11 on atypical and 10 on typical)	NAA, Cho and Cr, illness duration, duration on atypical antipsychotic medication, WCST	Patients receiving typical antipsychotics had lower NAA concentration than patients receiving atypical antipsychotics. NAA concentration was inversely correlated with illness duration and positively with duration on atypical antipsychotic medication. NAA concentration was inversely correlated with WCST perseverative errors.
Goff et al. (2002)	Schizophrenia patients (*n* = 17; of whom 10 switched from conventional to olanzapine and had MRS at baseline and 8-week follow-up).	Glu/Cr ratio, SANS	Glu/Cr ratio was increased by 12.5% (non-significant) following switch to olanzapine. Patients who improved on SANS symptoms had greater Glu/Cr ratio (45.8%) compared to patients who deteriorated on SANS symptoms (20.8%). No significant correlation between SANS total and change in Glu/Cr ratio after switch.
Yamasue et al. (2002)	Schizophrenia patients (*n* = 15; all on typical) Healthy participants (*n* = 13)	NAA, Cho and Cr; grey matter volume; age, height, body weight, socio-economic status, parental socio-economic status, years of education, PANSS, onset of illness, illness duration, antipsychotic medication dose.	Patients had lower NAA/Cho ratio and greater Cho/Cr ratio compared to healthy participants after controlling for grey matter volume. NAA/Cho ratio was positively correlated with grey matter volume in patients, not healthy participants. NAA/Cho ratio was inversely correlated with PANSS blunted affect score.
Theberge et al. (2004, 2002)	First-episode neuroleptic naïve schizophrenia patients (*n* = 21) Healthy participants (*n* = 21)	*Analysis in Theberge 2002* NAA, Cho, Cr, Glu, Gln, mI in left ACC, SANS, SAPS *Analysis in Theberge 2004* NAA and Cho in left ACC DUP, DUI, DPS	*From Theberge 2002* Patients had greater Gln concentration compared to healthy participants. Metabolite concentrations were not correlated with SANS or SAPS. *From Theberge 2004* NAA concentration was positively correlated with DUP and inversely with DPS at trend level. Cho concentration was positively correlated with DUP.
Theberge et al. (2003)	Schizophrenia patients (*n* = 21; atypical *n* = 7, both *n* = 1, typical *n* = 13) Healthy participants (*n* = 21)	NAA, Cho, Cr, Glu, Gln, mI in left ACC, duration of illness	Glu and Gln concentrations were greater in patients than healthy participants. NAA concentration was inversely correlated with duration of positive symptoms.
Wood et al. (2007)	Schizophrenia male patients (*n* = 15; 13 atypical, 2 medication free) Healthy male participants (*n* = 14)	NAA and Glx in dorsal and rostral regions, PANSS five factors, illness duration	Patients had lower NAA concentration compared to healthy participants. Right dorsal Glx was inversely correlated with illness duration.
Ohrmann et al. (2008)	Schizophrenia patients (*n* = 43, all on atypical) Healthy participants (*n* = 37)	NAA, Cho, Cr, Glx, WCST learning potential	No difference in metabolite concentration between patients and healthy participants. NAA and Glx concentrations were positively associated with WCST learning potential
Sanches et al. (2008)	Schizophrenia patients (*n* = 37) Healthy participants (*n* = 37)	NAA/(Cho + Cr) ratio in left and right anterior middle frontal gyrus, ACC and perirolandic area (reference), basal skin conductance and skin conductance response to auditory stimuli of 1 s tones of 80 dB. Patients and healthy participants classified into skin conductance responders (2 S.D. below mean skin conductance in healthy participants) and non-responders.	NAA/(Cho + Cr) ratio was lower in patients than healthy participants in the anterior middle frontal gyrus and ACC bilaterally. NAA/(Cho + Cr) ratio did not differ between skin conductance response groups based on skin conductance response to auditory stimuli. Basal skin conductance non-responders patients had lower NAA/(Cho + Cr) ratio than healthy participants in left and right ACC.
Tayoshi et al. (2009)	Long-term schizophrenia (*n* = 30; antipsychotic medication type not reported) Healthy participants (*n* = 25)	NAA, Cho, Cr, Glu, Gln, mI Gender, PANSS total and subscales, duration of illness, duration of therapy, antipsychotic dosage, benzodiazepine dose	Patients had lower Glu and mI concentration compared to healthy participants. Male patients had lower NAA, Cr, Glu and mI concentration than male healthy participants. Males had greater Gln concentration compared to females. Metabolite concentration was not correlated with symptoms or other clinical variables.
Ongur et al. (2009)	Schizophrenia patients (*n* = 15) Bipolar disorder patients (*n* = 15) Healthy participants (*n* = 22)	Cr in ACC (hypothesized area) and parietal-occipital cortex (reference area), MADRS, PANSS, YMRS	Patients had lower Cr than healthy participants in both ACC and parietal-occipital cortex. No significant correlations between Cr concentration and clinical measures.

ACC: anterior cingulate cortex, Cho—Choline, Cr—Creatine/Phosphocreatine, DPS—duration of psychotic symptoms, DUI—duration of untreated illness, DUP—duration of untreated psychosis, Gln—Glutamine, Glu—Glutamate, Glu/Cr ratio, SANS—Schedule for the Assessment of Negative Symptoms, Glx—Glutamate + Glutamine, MADRS—Montgomery Asberg Depression Rating Scale, mI—myo-Inositol, NAA: N-acetyl aspartate, PANSS—Positive and Negative Syndrome Scale, SAPS: Schedule for the Assessment of Positive Symptoms, WCST—Wisconsin Card Sorting test, YMRS—Young Mania Rating Scale.

**Table 1:** Battery of neuropsychological measures.

Neuropsychological test	Variables used in analysis
*Executive function*	
Brixton Test (Burgess and Shallice, 1997)	Perseverative errors
Wisconsin Card Sorting Test (WCST) (Heaton et al., 1993)	Perseverative errors

*Working/short-term memory*	
Letter number test (Gold et al., 1997)	Total number of items correct
Wechsler Memory Scale—Revised (WMS-R) Logical Memory (Wechsler, 1987)—Immediate recall	Unit recall scaled score
Hopkins Verbal Learning Test (Shapiro et al., 1999)	Total number of items freely recalled

*Attention*	
Continuous Performance Test—identical pairs (Cornblatt et al., 1988)	Discriminability

*Emotional decision-making*	
Iowa Gambling Task (Bechara et al., 1994)	Overall learning (block 5 minus block 1)

**Table 2:** Demographic, clinical and neuropsychological characteristics of patients and healthy participants groups.

Characteristic	Patients	Healthy participants	Test	*χ*^2^ (*df*)	*P*
Gender: male/female (*n*)	24/6	13/2	Chi-squared	0.304 (1)	0.581
	mean, S.D.	mean, S.D.		*F* or *z* (*df*)	
Age in years	36.40, 8.64	35.40, 13.34	ANOVA	0.092 (1,43)	0.763
Years in education	13.83, 2.15	15.61, 3.23	ANOVA	4.555 (1,41)	0.039
Illness duration, years	12.98 8.94				

*Symptoms (PANSS)*					
Positive	16.93, 3.95				
Negative	17.87, 4.52				
General psychopathology	31.70, 6.39				
Total	66.50, 13.17				
Medication level (chlorpromazine equivalents)	502.67, 327.88				
Medication type (*n*)					
Olanzapine	12				
Clozapine	6				
Risperidone	5				
Aripiprazole	2				
Flupentixol	2				
Amisulperide	1				
Haloperidol	2				

*Neuropsychological function*					
Brixton perseverative errors	0.50, 0.78	0.31, 0.48	Mann–Whitney *U*	− 0.552 (1,41)	0.648
WCST perseverative errors	18.17, 8.86	11.75, 7.51	ANOVA	4.870 (1,40)	0.033
Letter number test number correct	13.80, 3.80	16.92, 2.96	ANOVA	6.925 (1,41)	0.012
WMS logical memory immediate unit recalled	7.10, 2.58	9.69, 3.33	ANOVA	7.533 (1,40)	0.009
Hopkins total freely recalled	21.86, 5.38	26.92, 5.17	ANOVA	8.122 (1,40)	0.007
CPT discriminability	0.29, 0.59	1.87, 1.00	ANOVA	14.257 (1,39)	0.001
IGT overall learning	2.80, 11.83	8.92, 14.73	ANOVA	2.092 (1,41)	0.156

**Table 3:** ACC metabolite and grey matter concentrations (mean, S.D.) at baseline at each voxel in patients and healthy participants.

CSI variable (millimolars)	Patient (*n* = 30)	Healthy participants (*n* = 15)	*F* (*p*)
NAA	7.42, 1.26	8.56, 1.39	7.682 (0.008)
Cho	1.90, 0.38	1.99, 0.36	0.654 (0.423)
Cr	7.03, 1.24	7.89, 1.02	5.345 (0.026)
Grey matter	53.99, 10.51	61.39, 8.58	5.549 (0.023)

**Table 4:** Clinical correlates of metabolite concentration at baseline (patients only).

		NAA concentration		Cho concentration		Cr concentration	
	*r* or *ρ*	*r* (*P*)	*r*_p_^1^ (*P*)	*r* (*P*)	*r*_p_^1^ (*P*)	*r* (*P*)	*r*_p_^1^ (*P*)
Illness duration	*r*	− 0.059 (0.756)	0.091 (0.640)	− 0.064 (0.735)	0.045 (0.817)	− 0.291 (0.118)	− 0.244 (0.203)
Antipsychotic medication level	Rho	0.197 (0.298)		0.442 (0.014)		0.073 (0.700)	
Symptoms (PANSS)							
Positive (P) symptoms	*r*	− 0.489 (0.006)	− 0.430 (0.020)	− 0.391 (0.033)	− 0.325 (0.085)	− 0.308 (0.098)	− 0.262 (0.170)
Negative (N) symptoms	*r*	− 0.297 (0.111)	− 0.418 (0.024)	− 0.287 (0.124)	− 0.366 (0.051)	− 0.211 (0.264)	− 0.262 (0.170)
General psychopathology (GP)	*r*	− 0.416 (0.022)	− 0.506 (0.005)	− 0.299 (0.109)	− 0.341 (0.070)	− 0.204 (0.281)	− 0.219 (0.254)
Total	*r*	− 0.450 (0.013)	− 0.512 (0.004)	− 0.361 (0.050)	− 0.384 (0.040)	− 0.263 (0.160)	− 0.266 (0.163)
Individual symptoms							
Delusions (P)	Rho	− 0.411 (0.024)		− 0.342 (0.064)		− 0.192 (0.309)	
Disorganisation (P)	Rho	− 0.525 (0.003)		− 0.369 (0.045)		− 0.136 (0.475)	
Persecution (P)	Rho	− 0.390 (0.033)		− 0.193 (0.307)		− 0.068 (0.719)	
Hostility (P)	Rho	0.062 (0.745)		0.370 (0.044)		0.163 (0.390)	
Passive social withdrawal (N)	Rho	− 0.370 (0.044)		− 0.393 (0.032)		− 0.261 (0.163)	
Stereotyped thinking (N)	Rho	− 0.422 (0.020)		− 0.073 (0.684)		− 0.221 (0.240)	
Somatic concern (GP)	Rho	− 0.419 (0.021)		− 0.407 (0.026)		− 0.129 (0.496)	
Preoccupation (GP)	Rho	− 0.528 (0.003)		− 0.201 (0.287)		− 0.264 (0.156)	

*r*_p_^1^: partial correlation controlling for GM concentration; P: PANSS positive symptom subscale; N: PANSS negative symptom subscale; GP: PANSS general psychopathology subscale.

**Table 5:** Neuropsychological correlates of metabolite concentration at baseline in patients and healthy participants.

Neuropsychological function	Group	*N*	*r* or rho	NAA concentration		Cho concentration		Cr concentration	
				*r* or rho	*r*_p_^1^ (*P*)	*r* or rho	*r*_p_^1^ (*P*)	*r* or rho	*r*_p_^1^ (*P*)
Brixton perseverative errors	Patient	30	Rho	0.180 (0.341)		− 0.198 (0.293)		0.331 (0.074)	
	Control	13	Rho	0.356 (0.232)		0.356 (0.232)		0.579 (0.038)	
WCST perseverative errors	Patient	30	*r*	− 0.097 (0.610)	− 0.139 (0.473)	− 0.011 (0.956)	− 0.030 (0.877)	− 0.102 (0.592)	− 0.116 (0.550)
	Control	12	*r*	0.271 (0.394)	0.248 (0.461)	0.390 (0.211)	0.368 (0.265)	− 0.225 (0.483)	0.312 (0.35)
Letter–number test number correct	Patient	30	*r*	0.303 (0.104)	0.379 (0.043)	− 0.163 (0.388)	− 0.160 (0.407)	− 0.130 (0.493)	− 0.124 (0.523)
	Control	13	*r*	0.557 (0.048)	0.529 (0.077)	0.011 (0.971)	− 0.083 (0.797)	0.156 (0.611)	− 0.030 (0.927)
WMS logical memory immediate unit recalled	Patient	29	*r*	0.301 (0.112)	0.331 (0.085)	0.054 (0.781)	0.045 (0.822)	− 0.017 (0.932)	− 0.025 (0.898)
	Control	13	*r*	0.330 (0.271)	0.278 (0.382)	− 0.453 (0.120)	− 0.610 (0.035)	− 0.022 (0.946)	− 0.267 (0.401)
Hopkins verbal learning test total number freely recalled	Patient	29	*r*	0.127 (0.512)	0.119 (0.564)	0.181 (0.348)	0.177 (0.369)	0.269 (0.159)	0.265 (0.173)
	Control	13	*r*	0.576 (0.039)	0.555 (0.061)	− 0.173 (0.572)	− 0.330 (0.295)	0.277 (0.36)	0.061 (0.851)
CPT discriminability	Patient	29	*r*	− 0.037 (0.848)	0.158 (0.423)	− 0.114 (0.555)	0.016 (0.936)	0.003 (0.986)	0.088 (0.656)
	Control	12	*r*	0.329 (0.297)	0.275 (0.413)	− 0.209 (0.514)	− 0.346 (0.297)	0.207 (0.519)	0.006 (0.986)
IGT overall learning	Patient	30	*r*	0.179 (0.343)	0.210 (0.273)	0.026 (0.891)	0.029 (0.879)	0.062 (0.747)	0.064 (0.742)
	Control	13	*r*	0.042 (0.892)	0.078 (0.811)	− 0.063 (0.839)	− 0.025 (0.939)	− 0.225 (0.461)	− 0.169 (0.600)

*r*_p_^1^: partial correlation controlling for GM concentration.

**Table 6:** Demographic, clinical and metabolite concentration characteristics at baseline of CBTp + SC (*n* = 11), SC control (*n* = 13), and SC responsive (*n* = 6) patients.

Characteristic	CBTp + SC	SC control	SC responsive	*χ*^2^	*P*
Gender: male/female (*n*)	6/5	12/1	6/0	7.185	0.028
	mean, S.D.	mean, S.D.			
Age in years	34.64, 8.62	37.46, 8.77	37.33, 9.42	0.745	0.689
Years in education	14.45, 2.46	13.46, 1.76	13.77, 2.4	1.156	0.561
Illness duration, years	11.94, 8.60	13.77, 9.70	13.17, 9.28	0.303	0.860
Symptoms (PANSS)					
Positive	17.45, 2.95	19.15, 2.64	11.17, 1.72	14.644	0.001
Negative	18.18, 4.98	19.23, 3.86	14.33, 3.61	5.779	0.056
General psychopathology	32.27, 6.75	34.0, 5.49	25.67, 3.83	6.775	0.034
Total	67.91, 12.87	72.39, 9.94	51.17, 7.78	9.512	0.009
Medication level	564.42, 303.17	500.55, 394.78	394.05, 207.59	1.787	0.409
Metabolite concentration					
NAA	7.65, 0.92	6.85, 1.43	8.23, 0.91	5.313	0.070
Cho	1.92, 0.60	1.86, 0.24	1.94, 0.18	0.493	0.781
Cr	6.89, 1.40	6.87, 0.95	7.48, 1.60	1.047	0.592
Grey matter	57.66, 10.71	51.17, 11.36	53.37, 7.14	1.096	0.578

**Table 7:** Symptoms, metabolite and grey matter concentration (mean, S.D.) at baseline and follow-up in CBTp + SC and SC control groups.

CSI variable	Baseline	CBTp + SC (*n* = 7)	*z* (*P*)	Baseline	SC control (*n* = 4)	z (*P*)
		FOLLOW-UP			FOLLOW-UP	
Clinical symptoms						
Positive symptoms	18.14 (2.34)	15.67 (3.50)	− 2.032 (0.042)	19.25 (2.22)	18.25 (2.63)	− 0.365 (0.715)
Negative symptoms	18.57 (5.71)	15.67 (2.34)	− 0.813 (0.416)	18.75 (4.99)	22.00 (6.16)	− 1.841 (*0.066*)
General psychopathology	32.00 (5.66)	27.33 (4.97)	− 1.382 (0.167)	35.00 (6.68)	32.75 (5.91)	− 0.535 (0.593)
Total symptoms	68.71 (12.27)	58.67 (10.17)	− 1.472 (0.141)	73.00 (10.42)	73.00 (13.64)	− 0.001 (1.000)
Metabolite concentration						
NAA	7.65 (0.68)	8.31 (0.77)	− 1.859 (*0.063*)	7.40 (0.69)	7.04 (0.38)	− 1.826 (0.068)
Cho	1.85 (0.42)	1.93 (0.2)	− 0.507 (0.612)	1.90 (0.18)	2.01 (0.08)	− 0.730 (0.465)
Cr	6.79 (0.89)	7.25 (1.03)	− 0.845 (0.398)	7.02 (0.16)	7.36 (0.99)	− 0.730 (0.465)
Grey matter	57.06 (11.55)	58.60 (8.51)	− 0.338 (0.735)	53.53 (5.49)	53.24 (3.35)	− 0.365 (0.715)

## Appendix B Supplementary Data

Supplementary Table Correlation between metabolite concentration at baseline and residual symptom change following CBTp.
