# Erratum to “N-acetyl aspartate concentration in the anterior cingulate cortex in patients with schizophrenia: A study of clinical and neuropsychological correlates and preliminary exploration of cognitive behaviour therapy effects” [Psychiatry Research: Neuroimaging 182(3) (2010) 251–260]

**DOI:** 10.1016/j.pscychresns.2011.09.015

**Published:** 2011-12-30

**Authors:** Preethi Premkumar, Vivek A. Parbhakar, Dominic Fannon, David Lythgoe, Steven C. Williams, Elizabeth Kuipers, Veena Kumari

**Affiliations:** a Department of Psychology, Institute of Psychiatry, King's College London, London, United Kingdom; b Division of Psychological Medicine and Psychiatry, Institute of Psychiatry, King's College London, London, United Kingdom; c Centre for Neuroimaging Sciences, Institute of Psychiatry, King's College London, London, United Kingdom; d NIHR Biomedical Research Centre for Mental Health, South London and Maudsley NHS Foundation Trust, London, London, United Kingdom

The authors regret that the above mentioned paper contained a serious error relating to Fig. 1 of the original article.

**Fig. 1:**
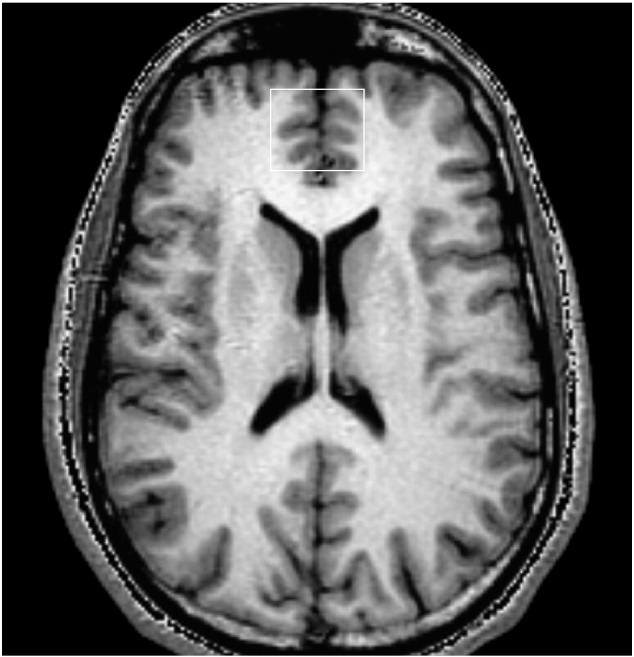
An SPGR image taken of a healthy participant that shows the position of the magnetic resonance spectroscopy box used to define the anterior cingulate cortex.

The authors apologize for using the figure without permission and not acknowledging the source of the figure, which was taken from the article by Yamasue et al. (2002). The figure was not linked to its proper source due to an unfortunate chain of events. It was originally included as an exemplar as part of the training given on the procedure for extracting the CSI data. Dr Virginia Ng, who had finalised the region-of-interest positioning for this study and conducted the training program, unfortunately passed away suddenly towards the end of the study in January 2008. Later, it was erroneously taken as implicit that the training image used to delineate the region of interest was derived from a participant in our own study, as opposed to having been taken from an earlier publication.

To correct the error in our publication, we have supplied an image of an SPGR scan depicting the axial slice from which the anterior cingulate cortex was measured using a control participant in our study (Fig. 1). The details of how the SPGR image was used to define the magnetic resonance spectroscopy (MRS) box are as follows. The genu of the corpus callosum was located on the midline sagittal localiser image. The SI coordinates of the point 8 mm above the most anterior portion of the genu of the corpus callosum was noted, and the axial slice of the oblique-axial SPGR closest to this position was displayed. This image was used to graphically prescribe a rectangular region for PRESS localised CSI. The centre of this slice would be the centre of the PRESS excited volume in the SI direction. The position and size of the rectangle (approximately 3 cm × 3 cm) were adjusted to cover most of the grey matter in the anterior cingulate cortex, avoiding the scalp, frontal sinuses and ventricles. The position of the box was also checked on the slices above and below to ensure correct placement of the whole 1.5 cm thick PRESS excited volume.

The same SPGR that was used to prescribe the CSI was segmented into grey matter, white matter and CSF fractions using SPM-2. The fractions of the different tissue types were determined for each CSI voxel using automated routines in SAGE/IDL (GE, Waukesha, WI). The positions of the CSI voxels and PRESS-localised volume relative to the SPGR were extracted automatically from the CSI and SPGR file headers. This assumes that the subject does not move between the SPGR and the MRSI acquisitions; therefore the time between the two scans was kept to a minimum. A similar routine in SAGE/IDL was also used to extract the raw data for each MRSI voxel to pass to LC model for analysis. The routine allows large 2D-MRSI datasets to be analysed and production of quantitative metabolite maps using the frequency-domain fitting method LCModel (Provencher, 1993; McLean et al., 2000).

The authors greatly regret the error, and are grateful for the understanding of Professor H. Yamasue and colleagues, the Editor of *NeuroReport*, and its publisher (Wolters Kluwer Health Medical Research/Lippincott Williams & Wilkins).
