# Supplementary material for: N-acetyl aspartate concentration in the anterior cingulate cortex in patients with schizophrenia: A study of clinical and neuropsychological correlates and preliminary exploration of cognitive behaviour therapy effects
Source: Psychiatry Res. 2010 Jun 30;182(3):251–60. doi: 10.1016/j.pscychresns.2010.02.008 (PMC4024608; doi:10.1016/j.pscychresns.2010.02.008)
Supplement: Supplementary Table — Correlation between metabolite concentration at baseline and residual symptom change following CBTp. [file mmc1.pdf]

Supplementary Table. Correlation between metabolite concentration at baseline and residual symptom change following CBTp.

**CSI variable**	**CBTp+SC (n=8)**		
	**NAA**	**Cho**	**Cr**
	**rho (p)**	**rho (p)**	**rho (p)**
Positive symptoms	0.357 (0.385)	0.310 (0.456)	<0.001 (1.000)
Negative symptoms	-0.262 (0.531)	0.190 (0.651)	-0.167 (0.693)
General psychopathology	-0.252 (0.548)	0.611 (0.108)	0.228 (0.588)
Total symptoms	-0.071 (0.867)	0.238 (0.570)	-0.071 (0.867)
